# MEF2A Promotes Keratinocyte Proliferation and Migration Under High‐Glucose Conditions via Activation of the PI3K/AKT Signaling Pathway: Implications for Diabetic Wound Healing

**DOI:** 10.1002/jgm.70120

**Published:** 2026-11-05

**Authors:** Yuan Xue, Min Xuan, Linlin Feng, Jingwen Kuang, Bin Xu, Limin Wei, Pengcheng Xu

**Affiliations:** ^1^ Department of Thyroid and Head and Neck Oncology Surgery The First Affiliated Hospital, and College of Clinical Medicine of Henan University of Science and Technology Luoyang China; ^2^ Department of Burn and Plastic Surgery General Hospital of Southern Theater Command, PLA Guangzhou China; ^3^ The First Affiliated Hospital, and College of Clinical Medicine of Henan University of Science and Technology Luoyang China; ^4^ Oncology Medical Ward 2 The First People's Hospital of Pingdingshan Pingdingshan China; ^5^ Department of Breast Surgery The First Affiliated Hospital, and College of Clinical Medicine of Henan University of Science and Technology Luoyang China

**Keywords:** diabetes mellitus, keratinocyte, MEF2A, PI3K/AKT signaling pathway, wound healing

## Abstract

Delayed wound healing, a serious complication of diabetes, remains a clinical challenge. Myocyte enhancer factor 2A (MEF2A) functions in muscle‐specific and/or growth factor‐related transcription and is involved in cell growth, survival, and apoptosis. To evaluate the role of this transcription factor in keratinocytes during the diabetic wound healing process, we conducted a series of in vivo and in vitro experiments. We found that MEF2A expression was upregulated in skin wound tissues of a spontaneous type 2 diabetic db/db mouse model. Similarly, MEF2A was highly expressed in human keratinocytes (HaCaT) under high glucose (HG) conditions. In HaCaT cells, overexpression of MEF2A enhanced cell proliferation and migration under HG conditions and also effectively attenuated HG‐induced apoptosis, as evidenced by the upregulated anti‐apoptotic protein Bcl‐2 and the downregulated pro‐apoptotic protein Bax and caspase‐3 activity. Additionally, overexpression of MEF2A increased the phosphorylation level of AKT in HaCaT cells. However, treatment with the PI3K/AKT pathway inhibitor LY294002 reversed the beneficial effect of MEF2A on HaCaT proliferation and migration. Our study suggests that overexpression of MEF2A can activate the PI3K/AKT signaling pathway by enhancing the phosphorylation level of the Ser473 site of AKT protein in HaCaT cells, thereby regulating the proliferation and migration of HaCaT cells under HG conditions. This provides a potential therapeutic target for diabetic wound healing.

## Graphical abstract

MEF2A promotes keratinocyte function in diabetic wound healing by activating the PI3K/AKT signaling pathway. In vivo studies revealed an upregulation of MEF2A expression in the skin wound tissues of diabetic mouse models. In vitro functional experiments confirmed that under high‐glucose conditions, MEF2A overexpression activates the PI3K/AKT signaling pathway by enhancing the phosphorylation of AKT at the Ser473 residue. This activation ultimately promotes the proliferation and migration of human immortalized keratinocytes (HaCaT).

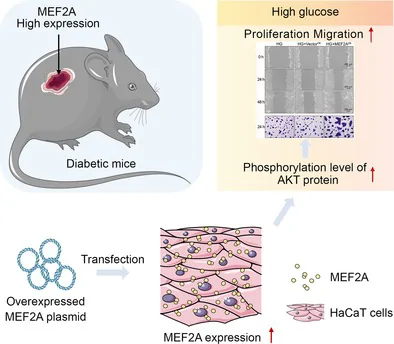

## 1 Introduction

Diabetes mellitus is a metabolic disorder characterized by insufficient insulin secretion or decreased sensitivity of target organs to insulin, arising from a combination of genetic and environmental factors [1]. It has emerged as a major public health issue posing a serious threat to global health [2]. The global prevalence of diabetes was estimated at 9.3% (affecting 463 million people) in 2019 and is projected to increase to 10.2% (578 million) by 2030 and 10.9% (700 million) by 2045 [3]. Chronic non‐healing wounds are among the most common complications of diabetes, affecting approximately 25% of diabetic patients [4]. These wounds are characterized by prolonged healing, difficult management, high cost, frequent recurrence, and significant rates of disability and mortality, all of which severely impair patients' quality of life and impose a substantial economic burden [5]. The mechanisms underlying impaired diabetic wound healing remain incompletely understood. Current clinical management often relies on local treatments such as mechanical debridement, revascularization, inflammation control, and offloading, yet therapeutic outcomes are frequently suboptimal [6]. Therefore, elucidating the molecular mechanisms of diabetic wound healing is crucial for developing novel strategies to promote the healing of refractory wounds.

Wound healing is a complex and dynamic process comprising three overlapping phases: inflammation, proliferation, and tissue remodeling [7]. Re‐epithelialization during the proliferative phase is a critical determinant of successful skin repair. This process depends on the functional activity of keratinocytes. Upon injury, keratinocytes are rapidly activated and contribute to tissue repair through behaviors such as proliferation, migration, differentiation, and matrix remodeling, processes coordinated via paracrine signaling with fibroblasts and immune cells [8, 9]. However, this healing process is significantly impaired in the diabetic state. Clinical studies have shown that the skin of diabetic patients often exhibits marked epidermal thinning [10], with keratinocytes demonstrating not only reduced proliferative capacity but also severely compromised migratory function [11]. The hyperglycemic environment induces physiological dysfunction in keratinocytes, characterized by dysregulated oxidative stress and inflammatory signaling, which hinders the re‐epithelialization process [12]. This impairment is considered a key factor contributing to delayed wound healing in diabetes. Consequently, therapeutically targeting enhanced keratinocyte proliferation and migration represents a potential strategy for improving diabetic wound healing.

MEF2A (myocyte enhancer factor 2A), a key member of the MADS‐box superfamily, is a crucial transcription factor [13]. It functions by forming homo‐ or heterodimers that bind specific response elements in the promoters of target genes, thereby regulating transcription involved in diverse cellular processes [14]. Initially identified as a core transcription factor in the development, differentiation, and metabolism of cardiac and skeletal muscle [15, 16], subsequent research has revealed that MEF2A's functions extend far beyond the muscular system. It is widely involved in regulating critical biological processes such as cell proliferation, migration, apoptosis, autophagy, and stress responses [17, 18]. For instance, under oxidative stress, MEF2A can delay cellular senescence by activating the PI3K/p‐AKT/SIRT1 pathway [19]. Overexpression of MEF2A also enhances cellular anti‐apoptotic capacity, accompanied by increased Bcl‐2 expression and decreased BAD expression [20]. Notably, knockdown of MEF2A under high glucose (HG) conditions significantly inhibits the proliferation and migration of cardiac fibroblasts [21], suggesting an important role for MEF2A in the context of aberrant glucose metabolism. Furthermore, MEF2A mRNA is highly expressed in skin tissue [22], and keratinocytes account for 90% of human epidermal cells. However, whether MEF2A regulates the functions of keratinocytes during the process of diabetic skin wound healing remains to be investigated.

This study aimed to explore the effects of MEF2A on the functions of keratinocytes under HG conditions and their underlying molecular mechanisms. Using a spontaneous type 2 diabetic db/db mouse skin wound model, we first determined the expression pattern of MEF2A in wound tissues. Furthermore, by establishing an MEF2A‐overexpressing model in human immortalized keratinocytes (HaCaT) under high‐glucose conditions, we systematically evaluated the effects of MEF2A on key cellular functions including proliferation, apoptosis, and migration, and explored the potential mechanisms involved. The primary objective was to investigate whether MEF2A can promote the proliferation of keratinocytes, inhibit apoptosis, and enhance their migratory capacity. This study contributes to uncovering the potential role of MEF2A in diabetic skin wound healing, offers a novel perspective for understanding its molecular mechanisms, and provides an experimental basis for exploring therapeutic strategies.

## 2 Materials and Methods

### 2.1 Establishment of Animal Models

Ten‐week‐old female spontaneous type 2 diabetic db/db mice (C57BLKS background, homozygous Lepr^db^ spontaneous mutation) and age‐matched female C57BL/6J wild‐type (WT) mice were used in this study. The db/db mice were purchased from Changzhou Cavens Laboratory Animal Co. Ltd. (Production License No. SCXK (Su) 2021‐0013), and C57BL/6J mice were provided by Jiangsu Huachuang Xinnuo Pharmaceutical Technology Co. Ltd. (Production License No. SCXK (Su) 2025‐0012). All mice were maintained in a specific pathogen‐free (SPF) barrier animal facility under a 12 h light/12 h dark cycle, with ad libitum access to sterile food and water as well as standard cage enrichment. After a 1‐week acclimatization period, the animals were used to establish the full‐thickness skin wound model. All animal procedures were approved by the Ethics Committee of The First Affiliated Hospital of Henan University of Science and Technology (Approval No. D‐2025‐B003) and were conducted in accordance with the National Institutes of Health Guide for the Care and Use of Laboratory Animals [23].

Prior to the procedure, the dorsal hair of each mouse was shaved, and the surgical skin area was thoroughly disinfected. Mice were anesthetized via isoflurane inhalation: 3% isoflurane (RWD, Shenzhen, China) was used for induction, and 2% isoflurane was maintained during the entire surgical procedure. Under stable anesthesia, two full‐thickness circular skin wounds (4 mm in diameter) were created on both sides of the dorsal midline using a skin biopsy punch. Six animals were included per experimental group (*n* = 6), and each data point in quantitative graphs corresponded to an individual mouse.

After wounding, random blood glucose levels were monitored at five time points (Days 0, 3, 7, 11, and 14 post‐wounding) to confirm persistent hyperglycemia throughout the wound healing process. At the same time points, photographs of the wounds were captured and the wound area was quantified. On Day 14 post‐wounding, all mice were euthanized via carbon dioxide (CO_2_) inhalation, and skin tissues from the edge of the wound, including both epidermis and dermis, were harvested. Each sample was divided into two portions: One portion was fixed and paraffin‐embedded for histological analysis (H&E staining) and immunohistochemistry, while the other portion was rapidly frozen in liquid nitrogen for subsequent RT‐qPCR and Western blot analysis.

### 2.2 H&E Staining and Immunohistochemical (IHC) Staining

Sections of 5 μm thickness were deparaffinized and rehydrated, followed by staining with hematoxylin (H8070, Solarbio) and eosin (A600190, Sangon). After dehydration, clearing, and mounting, the staining results were observed and photographed under a microscope.

The expression of MEF2A was detected via immunohistochemistry. Briefly, tissue sections were first deparaffinized and rehydrated. Antigen retrieval was then performed, followed by incubation with 3% hydrogen peroxide at 37°C for 15 min to block endogenous peroxidase activity. Subsequently, the sections were incubated with 1% BSA (A602440‐0050, Sangon) for 15 min for blocking, and then incubated overnight at 4°C with a 1:200 diluted MEF2A antibody (28819‐1‐AP, Proteintech). Afterward, the sections were incubated with a 1:100 diluted horseradish peroxidase (HRP)‐conjugated goat anti‐rabbit IgG (SE134, Solarbio) at room temperature for 45 min. Color development was carried out using a DAB substrate solution (C520017, Sangon), followed by counterstaining with hematoxylin (H8070, Solarbio). Staining results were then observed.

### 2.3 RNA Isolation and Quantitation

Total RNA was extracted from diabetic mouse skin, normal mouse skin, or HaCaT cells using TRIpure Reagent (RP1001, BioTeke), strictly following the manufacturer's protocol. The obtained RNA samples were subsequently reverse transcribed into cDNA using All‐in‐One First‐Strand cDNA Synthesis SuperMix (MD80101, Magen Biotechnology). Amplification reactions were performed on a RT‐qPCR system (Exicycler 96, BIONEER). The total reaction volume of 20 μL consisted of 1 μL cDNA, 0.5 μL of each primer, 0.3 μL SYBR GREEN I master mix (SR4110, Solarbio), and 10 μL of 2× Taq PCR MasterMix (BL1014, Biosharp). The reactions were performed under the following conditions: denaturation at 95°C for 5 min, followed by 40 cycles of denaturation at 95°C for 10 s, annealing at 60°C for 10 s and extension at 72°C for 15 s. Quantitative analysis of mRNA expression levels was conducted using the 2^−ΔΔCt^ method. The expression levels of mRNAs were normalized to β‐actin. RT‐qPCR primer information is shown in Table 1.

**TABLE 1:** RT‐qPCR primers.

Gene name	Forward (5′‐3′)	Reverse (5′‐3′)
homo MEF2A	TCAAGGCTTCAACTCGC	GGAACCCTGAGATAACTGC
homo β‐actin	TCAGGGTGAGGATGCCTCTC	CTCGTCGTCGACAACGGCT
mus Mea2f	GCAGTGCAAGTGGGATGTTG	CCCTTGCTTGATGGGGGAAT
mus β‐actin	GCCAGAGCAGTAATCTCCTTCT	AGTGTGACGTTGACATCCGTA

### 2.4 Cell Culture and Transient Transfection

Human HaCaT keratinocytes were purchased from Shanghai iCell Bioscience Inc (iCell‐h066), and were cultured in DMEM medium containing either 5 mM (normal glucose, NG) or 25 mM (HG) glucose.

HaCaT cells were transfected with the human MEF2A overexpression plasmid or the corresponding empty vector using Lipofectamine 3000 (L3000015, Invitrogen) following the manufacturer's protocol. The MEF2A overexpression plasmid was constructed on the pcDNA3.1 backbone, encoding the full‐length coding sequence (CDS) of human MEF2A (GenBank accession No. NM_001130926). This plasmid was obtained from YouBio Biological Co. Ltd. (China, Cat. No. G111770). After amplification and purification in our laboratory, the working stock of MEF2A overexpression plasmid used for transfection reached 271.5 ng/μL. The empty pcDNA3.1 vector from the same manufacturer was used as the negative control. Forty‐eight hours post‐transfection, MEF2A overexpression efficacy was evaluated by quantifying MEF2A mRNA levels via RT‐qPCR.

After 48 h of transfection, cells were cultured in medium containing 5 mM (NG) or 25 mM (HG) glucose. Following 24 h of NG or HG treatment, cells were collected for subsequent analysis. Caspase‐3 activity was tested using the caspase‐3 activity assay kit (BC3830, Solarbio).

In a separate experiment, cells were similarly transfected and treated with 20 μM LY294002 (L832989, Macklin) for 30 min at 48 h post‐transfection.

### 2.5 Cell Viability Assay

Cell viability was measured using the MTT Cell Proliferation and Cytotoxicity Detection Kit (KGA9301, KeyGEN). After the cells adhered to the surface, transfection and HG treatment were performed. Upon reaching the designated time points, 50 μL of MTT reagent was added to each well, and the cells were incubated at 37°C for 4 h. Afterward, the supernatant was carefully removed, and 150 μL of DMSO (KGH2110, KeyGEN) was added to each well to dissolve the formazan crystals. The absorbance of each well was measured at a wavelength of 490 nm.

### 2.6 Immunofluorescence (IF) Staining

The cells were fixed with 4% paraformaldehyde (80096618, Sinopharm), permeabilized with 0.1% (v/v) Triton X‐100 (ST795, Beyotime), and blocked with BSA (A602440‐0050, Sangon). Subsequently, the cells were incubated with primary antibodies, including anti‐Ki67 (A20018, Abclonal) anti‐p‐AKT (28731‐1‐AP, Proteintech), at 4°C overnight. This was followed by incubation with a Cy3‐conjugated goat anti‐rabbit IgG (SA00009‐2, Proteintech) secondary antibody at room temperature for 1 h. The nuclei were counterstained with DAPI (D106471, Aladdin). Finally, fluorescence images were acquired under a BX53 microscope (Olympus, Japan) fitted with a DP73 digital camera system (Olympus, Japan).

### 2.7 Cell Apoptosis Assay

Cell apoptosis was assessed using the annexin V‐fluorescein isothiocyanate (FITC)/propidium iodide (PI) apoptosis detection kit (BL107A, Biosharp). Briefly, cells were resuspended in 500 μL of binding buffer and stained with Annexin V‐FITC (5 μL) and PI (5 μL) for 15 min at room temperature in the dark. Apoptotic cells were then analyzed using a NovoCyte flow cytometer (Agilent) according to the manufacturer's instructions.

### 2.8 Transwell Assay and Wound Healing Assay

Cell migration was evaluated using transwell and wound healing assays. Briefly, cells from each group were digested with trypsin (T8150, Solarbio), and the digestion was terminated by adding complete culture medium. The cell suspension was collected and centrifuged at 150 g for 3 min, then washed with PBS and resuspended in serum‐free medium. A 200 μL aliquot of the cell suspension was added to the upper chamber of a transwell insert (14341, LABSELECT), while the lower chamber was filled with 800 μL of culture medium containing 10% FBS. The plates were incubated at 37°C under 5% CO_2_ for 24 h. After incubation, the transwell inserts were gently washed twice with PBS, fixed with 4% paraformaldehyde (C104188, Aladdin) at room temperature for 20 min, and stained with crystal violet solution (0528, Amresco) for 5 min. Excess stain was removed by rinsing with distilled water. The number of cells migrating to the lower side of the membrane was counted under an inverted microscope at 200× magnification.

Prior to the wound healing assay, the culture medium was replaced with serum‐free medium containing 10 μg/mL mitomycin C (HY‐13316, MCE) [24], and incubated for 1 h. To minimize variation in initial scratch width and ensure wound uniformity across all samples, all scratches were generated using sterile 200 μL pipette tips from the same batch. The scratching procedure was fully standardized: The operator applied consistent vertical pressure and scratched along pre‐marked straight lines at the bottom of each well to guarantee consistent scratch direction and width. After wounding, the cells were gently washed once with serum‐free medium to remove detached cells and debris, followed by incubation in a 37°C, 5% CO_2_ incubator. Images of the wound areas were captured at 0, 24 and 48 h under an inverted microscope. The migration distance was measured and compared among experimental groups.

### 2.9 Western Blot

Proteins were separated by SDS‐PAGE and transferred to polyvinylidene difluoride membranes. The membranes were blocked with 5% non‐fat milk in TBST (PR20011, Proteintech) at room temperature for 2 h and incubated overnight at 4°C with primary antibodies: anti‐MEF2A (A12059, Abclonal), anti‐Bax (A0207, Abclonal), anti‐Bcl‐2 (240122, Zenbio), anti‐p‐AKT^Ser473^ (AF0016, Affinity), anti‐AKT (A17909, Abclonal), or anti‐β‐actin (66009‐1‐Ig, Proteintech). Then the membranes were incubated with HRP‐conjugated goat anti‐mouse (SA00001‐1, Proteintech) or anti‐rabbit secondary antibodies (SA00001‐2, Proteintech) for 1 h at room temperature. Membranes were visualized by chemiluminescence reagent (PK10003, Proteintech).

### 2.10 Statistical Analyses

All statistical analyses were performed using GraphPad Prism 9.0. Quantitative data are presented as mean ± standard deviation (SD). For comparisons between two groups, an unpaired two‐tailed Student's *t*‐test was used when both normality and equal variance assumptions were met; if equal variance was violated, the unpaired *t*‐test with Welch's correction was applied. For comparisons among three or more groups at a single time point, one‐way ANOVA was performed, followed by Tukey's post hoc test for multiple pairwise comparisons. Experiments involving two independent variables (e.g., group and time) were analyzed using two‐way ANOVA, followed by Šidák's post hoc test. A *p*‐value < 0.05 was considered statistically significant. Unless otherwise stated, all in vitro experiments were independently repeated three times, with three technical replicates per experiment. For in vivo experiments, six mice per group were used, as indicated in the corresponding figure legends.

## 3 Results

### 3.1 Elevated MEF2A Expression in Skin Wound Tissues of Diabetic Mice

To investigate the expression pattern of MEF2A during diabetic wound healing, we established full‐thickness excisional skin wound models in WT C57BL/6J mice and spontaneous type 2 diabetic db/db mice. Only db/db mice with fasting blood glucose ≥ 16.7 mmol/L were enrolled to ensure a stable diabetic phenotype [25].

Consistent with the disease phenotype, db/db mice exhibited persistently elevated blood glucose throughout the observation period. As quantified in Figure 1A, glycemia in the db/db group remained stable at approximately 21–23 mmol/L from Day 0 to Day 14 post‐wounding, whereas WT mice maintained NG levels at around 7 mmol/L. The intergroup difference was statistically significant at all time points (all *p* < 0.0001), confirming sustained hyperglycemia during the wound repair process.

**FIGURE 1:**
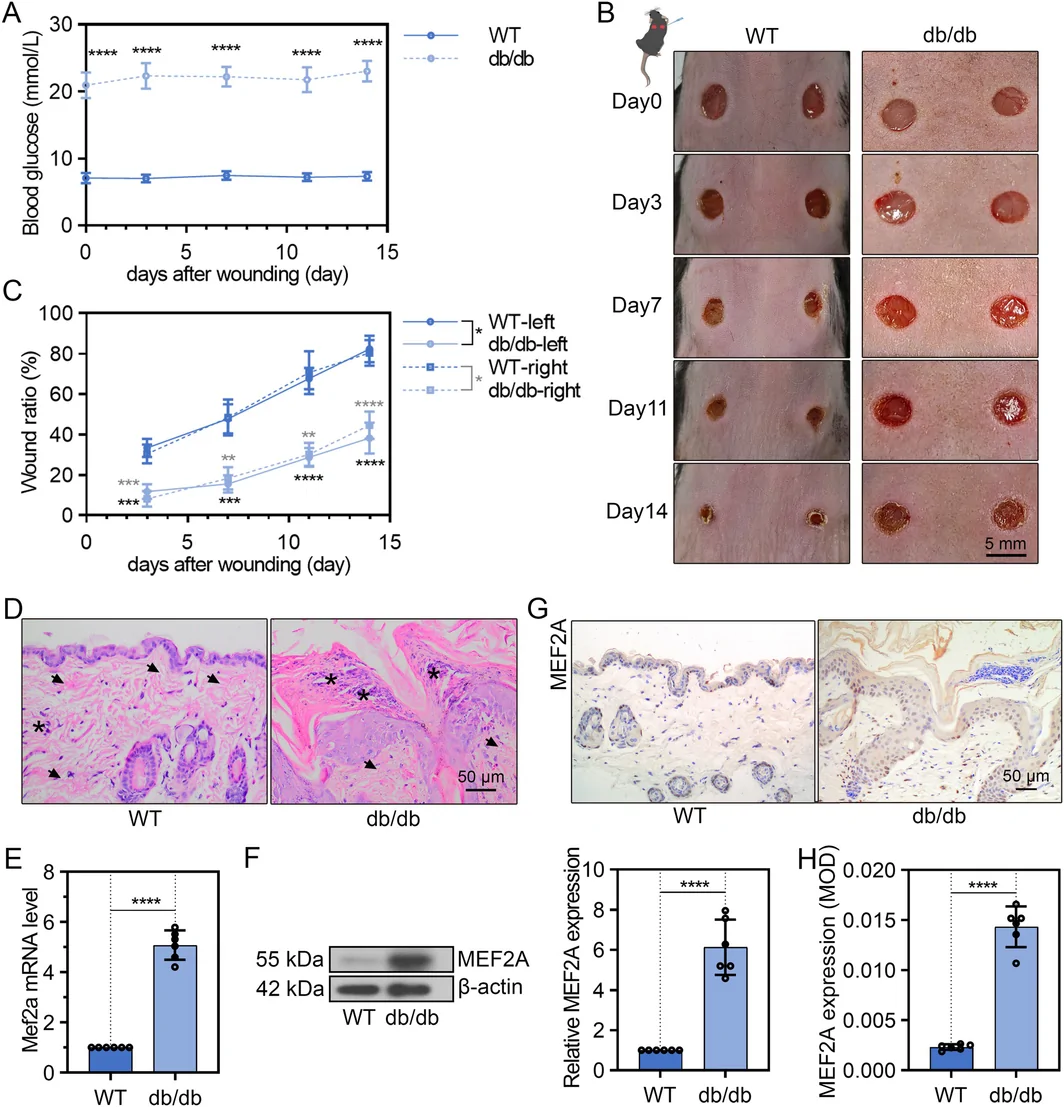
Elevated MEF2A expression in skin wound tissues of diabetic mice. (A) Blood glucose levels after wounding are presented as line graphs (*n* = 6). (B) Representative images of wounds over the healing course (*n* = 6). Scale bar: 5 mm. (C) Quantitative analysis of wound healing rates (*n* = 6). (D) Representative H&E‐stained images of wound tissues on day 14 post‐injury. Scale bar: 50 μm. Asterisks indicate infiltrating inflammatory cells; arrows indicate collagen fibers. (E) Relative mRNA expression of MEF2A in wound tissues measured by RT‐qPCR (*n* = 6). (F) Representative western blot analysis of MEF2A protein levels in wound tissues (*n* = 6). (G, H) IHC staining and quantitative results of MEF2A in wound tissues from WT and db/db mice (*n* = 6). Scale bar: 50 μm. WT: wild type mice; db/db: diabetic mice; Mef2a: myocyte enhancer factor 2A. *Mouse illustration in panel B adapted from Federico Claudi,* “Mouse Top Detailed,” *SciDraw.io (2026), available at* https://scidraw.io/drawing/mouse‐top‐detailed‐82f36ed0 *, licensed under CC BY 4.0 (* https://creativecommons.org/licenses/by/4.0/ *)*. Comparisons between two groups were performed using the *t*‐test. For time‐course experiments involving two independent factors (group and time), two‐way ANOVA was applied, followed by Šidák's post hoc test. Data are presented as mean ± SD. **p < 0.01, ***p < 0.001, ****p < 0.0001.

Monitoring results of wound morphology further revealed markedly delayed wound closure in db/db mice relative to WT controls (Figure 1B). Quantitative analysis of the wound area ratio showed that the wound closure rate was significantly lower in the db/db group from Day 3 onward, and the gap widened progressively over time (Figure 1C), demonstrating impaired cutaneous wound healing in the diabetic state. Histological evaluation by H&E staining on Day 14 post‐wounding further corroborated the impaired regenerative capacity in diabetic wounds (Figure 1D). In the WT group, the wound area exhibited a continuous and well‐stratified epidermis with neatly arranged cells, and the dermis showed densely organized collagen fibers (indicated by arrowheads) with minimal inflammatory cell infiltration. In contrast, the db/db group displayed a disrupted epidermal layer with disordered cell arrangement, accompanied by sparse and irregularly distributed collagen fibers and substantial inflammatory cell infiltration (indicated by asterisks). These histological features indicate that diabetic mice exhibit delayed tissue repair and a persistent inflammatory response, consistent with the macroscopic healing phenotype.

To determine whether MEF2A is associated with this impaired healing process, we examined its expression in wound tissues at both the transcriptional and translational levels. RT‐qPCR and Western blot analyses revealed that MEF2A expression was significantly upregulated in db/db mice compared with WT controls at both the mRNA and protein levels (Figure 1E,F). IHC staining further corroborated these findings and defined the spatial distribution of MEF2A in wound tissues (Figure 1G,H). In WT wounds, MEF2A immunoreactivity was predominantly confined to the epidermal layer with weak staining intensity and was barely detectable in the dermis. In contrast, db/db wound sections exhibited intense MEF2A‐positive signals widely distributed throughout both the epidermal and dermal layers. Semiquantitative analysis confirmed a significant increase in overall MEF2A expression in the db/db group.

Collectively, these results demonstrate that skin wound healing is significantly delayed in diabetic mice, accompanied by a marked elevation of MEF2A expression in wound tissues. These findings suggest a potential involvement of MEF2A in the pathophysiological processes underlying impaired diabetic wound healing.

### 3.2 Overexpression of MEF2A Promoted the Proliferation of HaCaT Cells Under HG Conditions

During the early stages of wound healing, the rapid proliferation of keratinocytes at the wound margin is essential for re‐epithelialization and restoration of the skin barrier [26, 27]. To investigate the role of MEF2A in diabetic skin wound healing, we utilized the human immortalized keratinocyte line (HaCaT) as an experimental model. This cell line is a well‐established tool in related research due to its stable phenotype and reliable reproducibility in studies of epidermal cell behaviors such as proliferation and migration [28, 29].

We first examined the effect of HG stress on MEF2A expression in HaCaT cells. Cells were cultured in either NG (5 mM) or HG (25 mM) medium for 24 h, followed by RT‐qPCR and Western blot analysis. The results showed that both mRNA and protein levels of MEF2A were significantly upregulated in the HG group compared with the NG group (Figure 2A,B). This finding was consistent with our observations in the animal model, suggesting that the upregulation of MEF2A may represent a compensatory mechanism activated in response to HG‐induced damage, aimed at maintaining cellular homeostasis.

**FIGURE 2:**
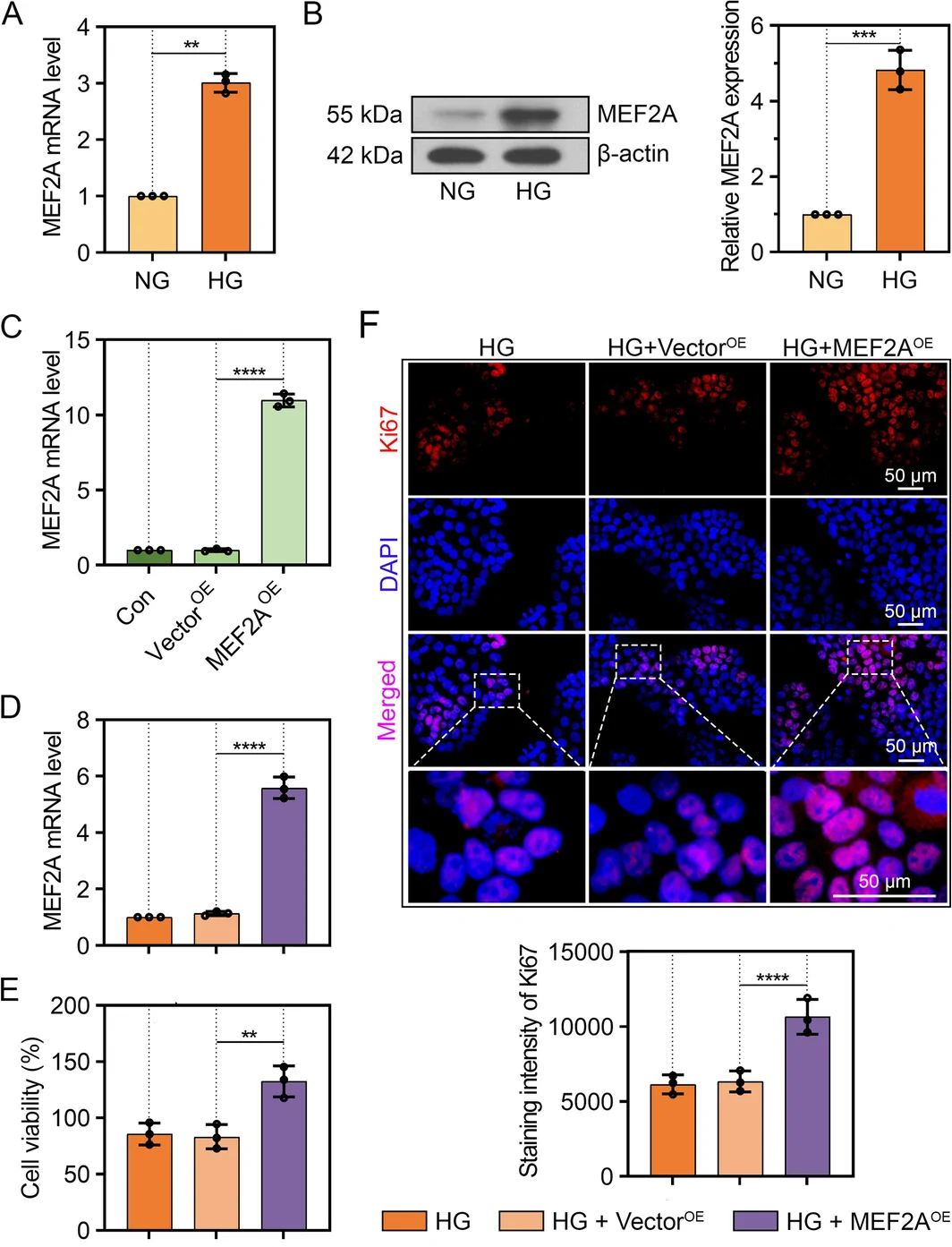
Effects of MEF2A on the proliferation of HaCaT cells under high glucose conditions. (A, B) MEF2A protein and mRNA levels in HaCaT cells cultured under normal glucose (5 mM) or high glucose (25 mM) conditions for 24 h (*n* = 3). (C) MEF2A mRNA expression in HaCaT cells transfected with the MEF2A overexpression plasmid, as detected by RT‐qPCR (*n* = 3). (D) Relative MEF2A mRNA levels in transfected HaCaT cells after 24 h of high‐glucose (25 mM) treatment, measured by RT‐qPCR (*n* = 3). (E) Cell viability assessed by MTT assay (*n* = 3). (F) IF staining and quantitative results of Ki67 (red) and nuclear counterstaining with DAPI (blue) (*n* = 3). Scale bar: 50 μm. NG: normal glucose; HG: high glucose; MEF2A^OE^: MEF2A eukaryotic overexpression plasmid; Vector^OE^: empty plasmid. Comparisons between two groups were performed using the *t*‐test, whereas comparisons among three or more groups were conducted using one‐way analysis of variance, followed by Tukey's post hoc test. Data are presented as mean ± SD. **p < 0.01, ***p < 0.001, ****p < 0.0001.

To further elucidate the direct regulatory role of MEF2A in cell proliferation, HaCaT cells were transfected with either an MEF2A overexpression plasmid (MEF2A^OE^) or an empty vector control (Vector^OE^). Untransfected HaCaT cells were set as baseline control. RT‐qPCR analysis at 48 h post‐transfection confirmed significantly elevated MEF2A mRNA levels in the MEF2A^OE^ group relative to the Vector^OE^ group (Figure 2C), verifying successful construction of the MEF2A‐overexpressing HaCaT cell model. Subsequent functional validation was performed under HG conditions. Both MEF2A^OE^ and Vector^OE^ cells were cultured in HG medium for 24 h. RT‐qPCR results confirmed elevated MEF2A expression in the HG + MEF2A^OE^ group compared with the HG + Vector^OE^ group (Figure 2D). MTT assays showed that cell viability in the HG + MEF2A^OE^ group was significantly higher than that in the HG + Vector^OE^ group, indicating that MEF2A overexpression enhanced the viability of HaCaT cells under HG conditions (Figure 2E). In parallel, under NG conditions, empty vector transfection did not cause significant changes in cell viability compared with untreated HaCaT cells; MEF2A overexpression slightly increased cell viability, although no statistically significant difference was observed (Figure S1A).

To determine if the enhanced viability was attributable to improved proliferative capacity, we assessed the expression of Ki67, a key marker of cell proliferation. IF staining demonstrated a significant increase in Ki67‐positive signals in MEF2A‐overexpressing cells under HG conditions compared with the control (Figure 2F). Under NG conditions, Ki67 staining intensity showed no significant difference between the untreated and empty vector groups; MEF2A overexpression induced a mild increase in Ki67 expression, but the difference did not reach statistical significance (Figure S1B,C).

Collectively, these results demonstrated that upregulation of MEF2A expression promotes proliferation and improves cell viability in keratinocytes under HG conditions.

### 3.3 MEF2A Inhibited Apoptosis of HaCaT Cells Under HG Conditions

Wound healing relies on the precise regulation of apoptosis at various stages; an imbalance in apoptosis (either excessive or insufficient) can impede the healing process [30]. In the case of diabetic wounds, hyperglycemia adversely affects cell differentiation and alters apoptotic pathways, which contribute to impaired healing [31].

To investigate the regulatory role of MEF2A in apoptosis under HG stress, we performed Annexin V/PI staining followed by flow cytometry. The results showed that compared with the HG + Vector^OE^ group, overexpression of MEF2A significantly reduced the proportion of apoptotic HaCaT cells (total early and late apoptotic cells) induced by HG (5.55% vs. 19.49%) (Figure 3A,B). In contrast, under NG conditions, the basal apoptosis rate of HaCaT cells was relatively low, and empty vector transfection did not cause significant changes in the proportion of apoptotic cells compared with untreated HaCaT cells. MEF2A overexpression slightly reduced the basal apoptosis level, though this difference did not reach statistical significance (Figure S1D). This indicates that the anti‐apoptotic effect of MEF2A is mainly targeted at HG‐induced cellular damage.

**FIGURE 3:**
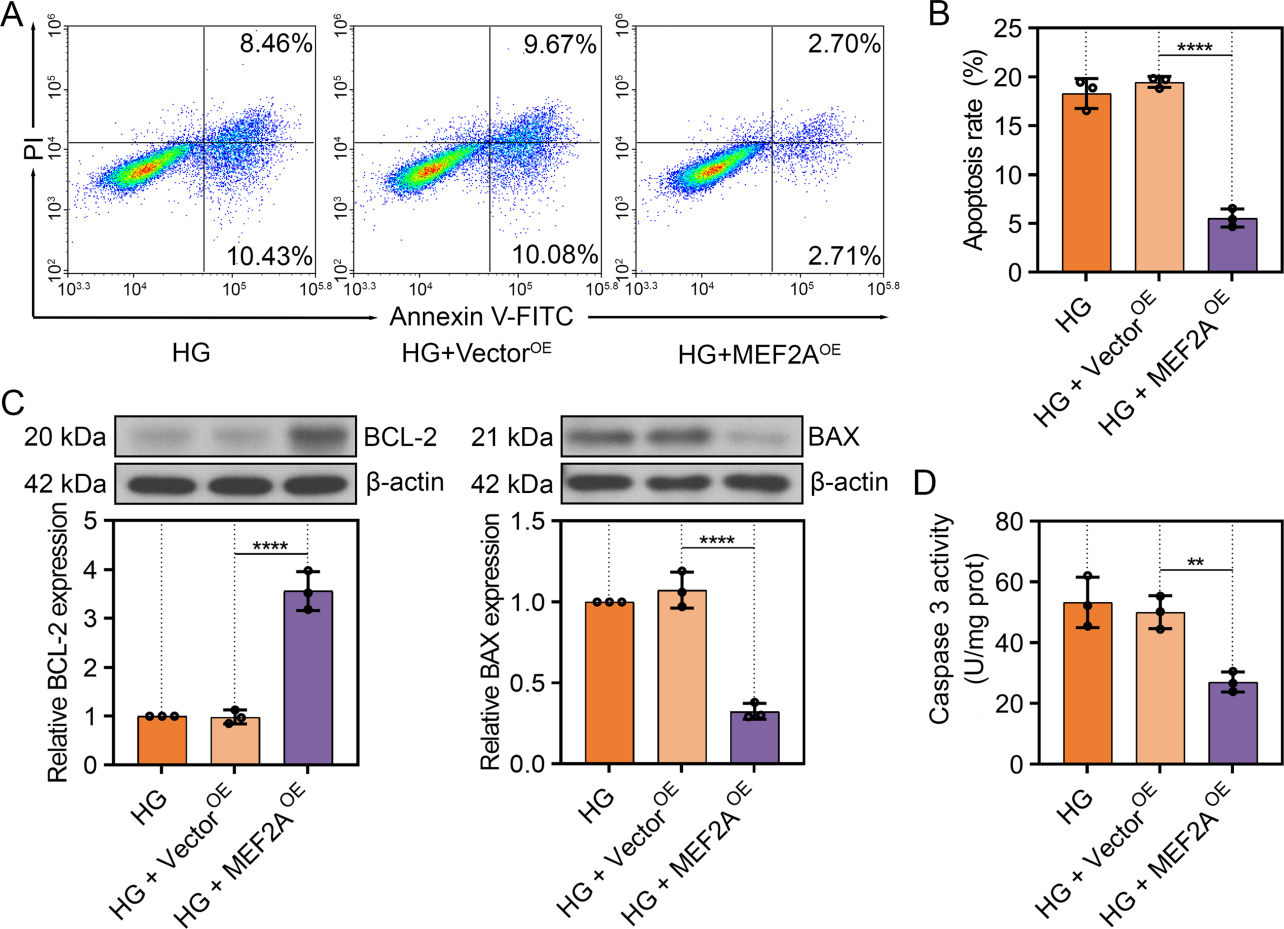
Effects of MEF2A on apoptosis in HaCaT cells under high glucose conditions. (A, B) Cell apoptosis was measured by flow cytometry (*n* = 3). (C) Representative western blot of BCL‐2 and BAX (*n* = 3). (D) Measurement of caspase 3 activities in HaCaT cells (*n* = 3). BCL‐2, B‐cell lymphoma‐2; BAX, BCL2‐Associated X protein. Comparisons among three or more groups were conducted using one‐way analysis of variance, followed by Tukey's post hoc test. Data are presented as mean ± SD. **p < 0.01, ****p < 0.0001.

To further elucidate the underlying molecular mechanism, we examined key proteins in the apoptotic pathway. Western blot analysis revealed that MEF2A overexpression significantly increased the expression of the anti‐apoptotic protein Bcl‐2 and decreased the expression of the pro‐apoptotic protein Bax under HG conditions (Figure 3C). In addition, MEF2A overexpression markedly reduced the activity of caspase‐3, a key effector molecule in the downstream apoptotic execution pathway (27.09 U/mg prot) (Figure 3D).

These findings indicated that MEF2A exerted a significant inhibitory effect on HG‐induced apoptosis in HaCaT cells by modulating the Bcl‐2 family proteins and suppressing caspase‐3 activity.

### 3.4 MEF2A Promoted the Migration of HaCaT Cells Under High‐Glucose‐Induced Injury

The directed migration of keratinocytes toward the wound center is a critical step for re‐epithelialization and ultimate wound closure [32, 33]. To investigate the effect of MEF2A on the migratory capacity of HaCaT cells under HG conditions, we performed cell scratch wound healing assays and transwell migration assays. The scratch assay revealed that after 24 h, the wound closure rate was significantly higher in the MEF2A‐overexpressing group compared with the HG + Vector^OE^ group (51.67% vs. 30.38%) (Figure 4A,B). Consistently, this pro‐migratory effect remained stable and significant at 48 h, with the MEF2A‐overexpressing group still showing a markedly higher wound closure rate than the control group (Figure 4B). Under NG conditions, empty vector transfection did not significantly affect the wound closure rate compared with untreated HaCaT cells; MEF2A overexpression slightly promoted cell migration at both 24 and 48 h, although no statistically significant differences were observed (Figure S1E). The transwell assay further demonstrated that MEF2A overexpression significantly increased the number of migrated cells (220 vs. 125) (Figure 4C,D).

**FIGURE 4:**
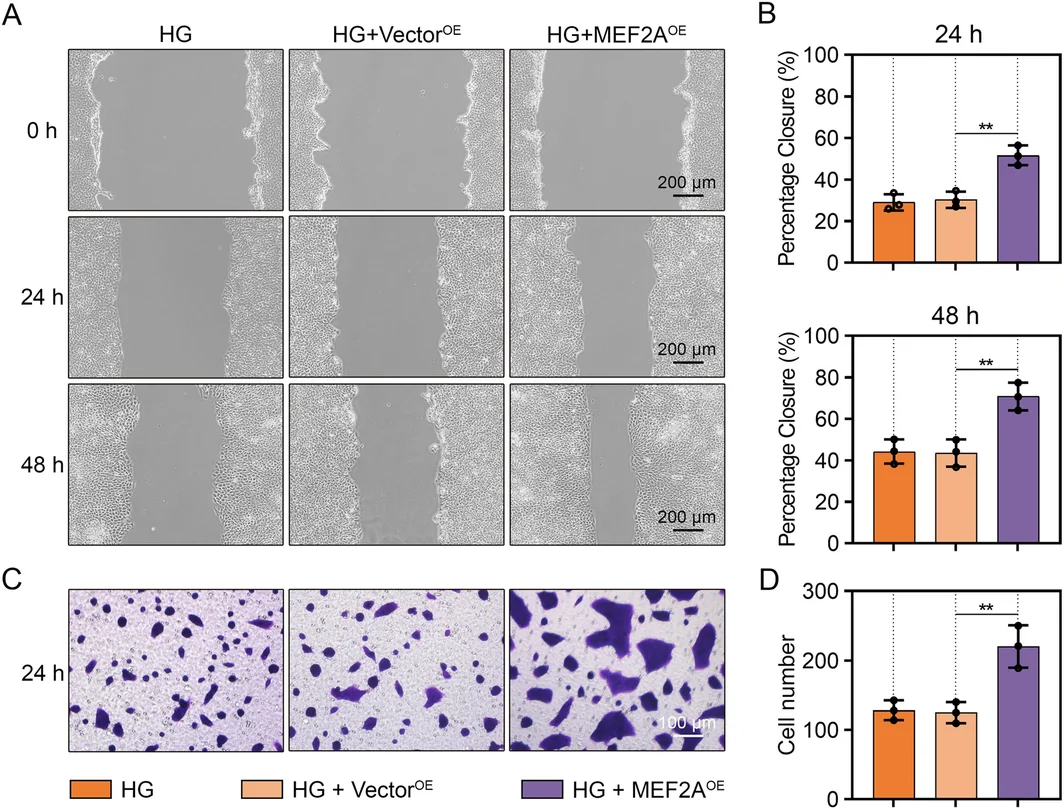
Effects of MEF2A on the migration of HaCaT cells damaged by high glucose. (A, C) Representative images from wound healing (A, scale bar: 200 μm) and transwell migration (C, scale bar: 100 μm) assays showing HaCaT cell migration after 24 or 48 h treatment under the indicated conditions. (B, D) Quantification of the wounded area closure rate (B) and number of migrating cells (D) (*n* = 3). Comparisons among three or more groups were conducted using one‐way analysis of variance, followed by Tukey's post hoc test. Data are presented as mean ± SD. **p < 0.01.

Collectively, these results indicated that upregulation of MEF2A significantly enhanced the migratory ability of HaCaT cells under HG conditions.

### 3.5 MEF2A Promoted the Proliferation and Migration of High‐Glucose‐Injured HaCaT Cells by Activating the PI3K/AKT Signaling Pathway

Although the critical role of MEF2A in regulating cell proliferation and migration has been established in this study, its downstream signaling mechanisms remain to be elucidated. Previous studies have suggested a potential link between MEF2A and the activation of the PI3K/AKT signaling pathway [21]. As a classic intracellular signaling cascade, the PI3K/AKT pathway plays a central regulatory role in cell proliferation, migration, survival, and metabolism [34]. Pathway activation is characterized by the phosphorylation of AKT, with phosphorylation at serine 473 (p‐AKT^Ser473^) serving as a key indicator of full AKT activation and a widely used marker for pathway activity [35].

Based on this, we examined the effect of MEF2A overexpression on AKT phosphorylation. Western blot analysis and corresponding quantification demonstrated that under HG conditions, MEF2A overexpression (HG + MEF2A^OE^ group) significantly increased p‐AKT^Ser473^ levels, whereas total AKT protein expression remained unchanged across groups (Figure 5A). This finding was further corroborated by IF staining for p‐AKT^Ser473^, which showed stronger fluorescence signals in both the cytoplasm and nucleus of the HG + MEF2A^OE^ group, and semiquantitative analysis confirmed a statistically significant difference (Figure 5B,C). These data indicate that MEF2A promotes AKT phosphorylation, but it was necessary to determine whether this pathway activation is essential for the observed biological functions of MEF2A.

**FIGURE 5:**
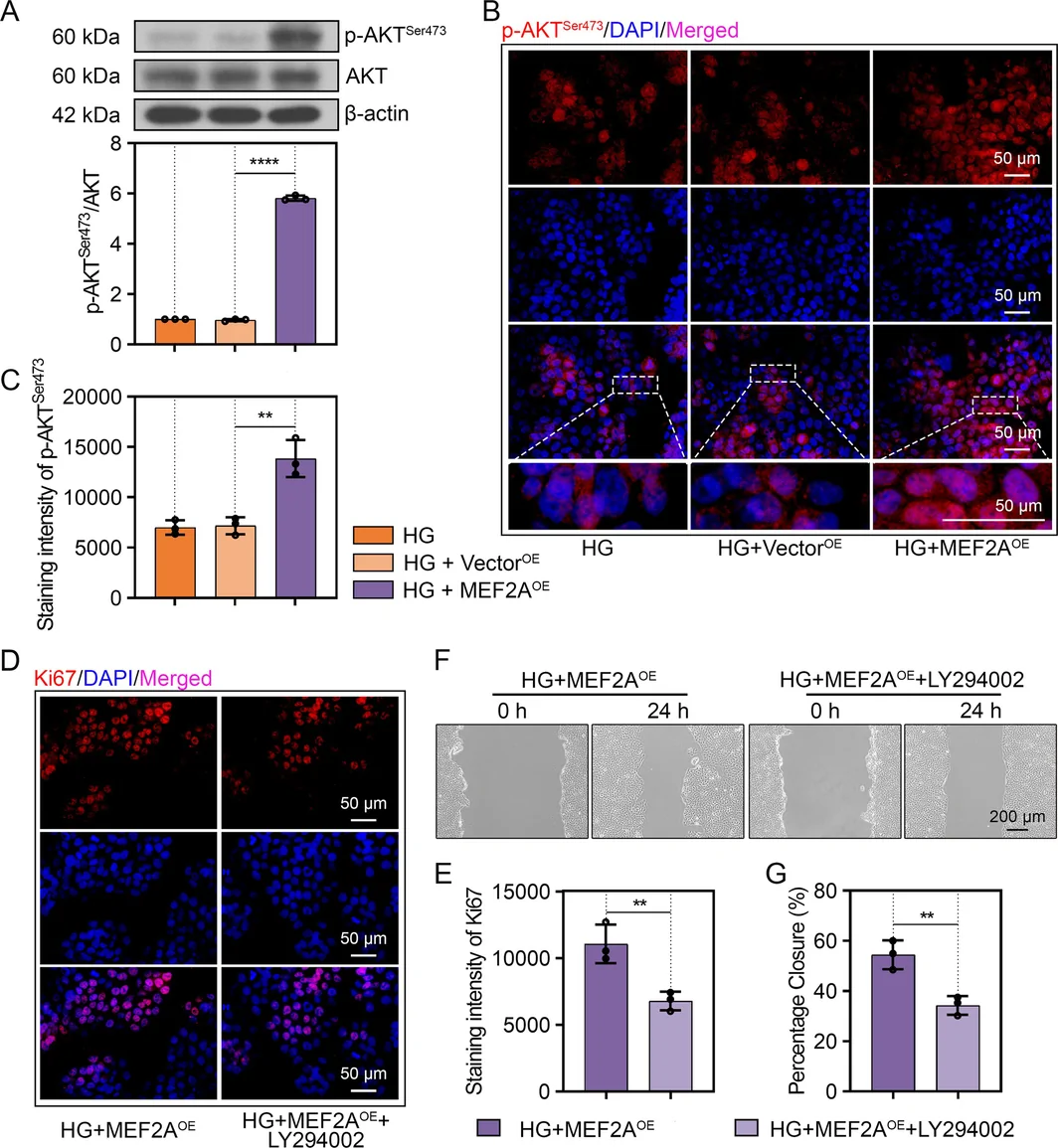
MEF2A regulated high‐glucose‐inhibited proliferation and migration of HaCaT cells via the PI3K/AKT pathway. (A) Representative western blot of p‐AKT^Ser473^, AKT (*n* = 3). (B, C) IF staining and quantitative results of p‐AKT^Ser473^ (red) and nuclei (DAPI, blue) (*n* = 3). Scale bar: 50 μm. (D, E) HaCaT cells were transfected and treated with 20 μM LY294002 for 30 min. IF staining and quantitative results of Ki67 (red) and nuclei (DAPI, blue) (*n* = 3). Scale bar: 50 μm. (F) Cell migratory capability assessed by wound healing assay. Scale bar: 200 μm. (G) Quantification of wounded area closure (*n* = 3). AKT, Protein kinase B; p‐AKT^Ser473^, AKT phosphorylated at the serine 473. Comparisons between two groups were performed using the *t*‐test, whereas comparisons among three or more groups were conducted using one‐way analysis of variance, followed by Tukey's post hoc test. Data are presented as mean ± SD. **p < 0.01, ****p < 0.0001.

To address this, we used the specific PI3K/AKT pathway inhibitor LY294002 to suppress pathway activity in the context of MEF2A overexpression. Assessment of proliferative status by Ki67 IF staining revealed that compared with the HG + MEF2A^OE^ group, the HG + MEF2A^OE^ + LY294002 group exhibited a significant reduction in Ki67 fluorescence intensity (Figure 5D,E), indicating that inhibition of the PI3K/AKT pathway reversed the pro‐proliferative effect induced by MEF2A overexpression. Furthermore, analysis of migratory capacity via the scratch wound healing assay showed that LY294002 treatment also significantly attenuated the enhanced migration mediated by MEF2A overexpression (Figure 5F,G). The wound closure rate of the HG + MEF2A^OE^ + LY294002 group at 24 h was significantly lower than that of the HG + MEF2A^OE^ group (34.38% vs. 54.55%).

In summary, as shown in the Graphical Abstract, this study demonstrated that MEF2A activated the PI3K/AKT signaling pathway by enhancing AKT phosphorylation at Ser473. More importantly, inhibition of this pathway with LY294002 significantly reversed the pro‐proliferative and pro‐migratory effects mediated by MEF2A overexpression. These results collectively indicated that the ability of MEF2A to improve keratinocyte functions under HG conditions was dependent on its regulation of the PI3K/AKT signaling pathway.

## 4 Discussion

Impaired wound healing is one of the most common and serious complications of diabetes, affecting approximately 25% of diabetic patients worldwide [36]. This condition leads to delayed wound closure, prolonged treatment, increased healthcare costs, and a significantly diminished quality of life, thereby posing a major public health challenge [37]. Although current clinical management primarily involves debridement, revascularization, inflammation control, and offloading therapy [36, 38, 39], the effectiveness of these approaches remains suboptimal. Therefore, there is an urgent clinical need to elucidate the molecular mechanisms underlying diabetic wound healing and to explore novel strategies for effective treatment.

This study focused on the role of MEF2A in diabetic wound healing. Our findings demonstrated that: (a) MEF2A expression was significantly upregulated in skin wound tissues of diabetic mice and in human immortalized keratinocytes (HaCaT) exposed to HG; (b) overexpression of MEF2A enhanced the proliferative capacity of HaCaT cells under HG conditions. Concurrently, it significantly suppressed HG‐induced apoptosis by downregulating the expression of pro‐apoptotic Bcl‐2 family proteins and reducing caspase‐3 activity. (c) MEF2A positively regulated the PI3K/AKT signaling pathway by increasing the phosphorylation level of AKT at Ser473, thereby effectively promoting the migratory capacity of HaCaT cells in an HG environment. In summary, our findings suggest that MEF2A may exert a protective role in diabetic wound healing and merit further investigation as a potential therapeutic target. To our knowledge, this study is the first to reveal the critical role of MEF2A in diabetic wound healing.

Wound healing is a highly coordinated dynamic process involving three overlapping phases: inflammation, proliferation, and remodeling [40]. The proliferative phase not only bridges the resolution of inflammation but is also an indispensable prerequisite for tissue remodeling; its efficiency directly determines the rate of granulation tissue formation and re‐epithelialization [41]. During this phase, various reparative cells, such as fibroblasts, vascular endothelial cells, and keratinocytes, are activated and collaborate to repair tissue defects through highly organized biological activities, including directed proliferation, migration, and synthesis/deposition of extracellular matrix (ECM) [42, 43]. Keratinocytes, as the primary functional cells of the epidermis, rely on efficient and coordinated proliferation and migration as the core driving force for re‐epithelialization [32, 33]. However, studies have shown that persistent hyperglycemia and concomitant insulin resistance can cause functional impairments in various cells, including keratinocytes (manifested as aberrant protein metabolism and endothelial dysfunction), thereby significantly compromising their proliferation, migration, and re‐epithelialization capacities [44]. Consequently, improving keratinocyte function is crucial for promoting diabetic wound healing. Our finding that MEF2A overexpression effectively enhances the physiological functions of keratinocytes under HG conditions—by promoting proliferation and migration while inhibiting apoptosis—provides important experimental evidence for exploring new strategies to improve diabetic wound healing.

The MEF2A gene is located on human chromosome 15q26.3. Its encoded protein belongs to the myocyte enhancer factor 2 (MEF2) family and functions primarily as a transcription factor within the nucleus [13]. Previous studies reported a critical role for MEF2A in cardiac and other muscle tissues and demonstrated that MEF2A knockdown inhibited HG‐induced proliferation and migration of cardiac fibroblasts by modulating the Akt and TGF‐β1/Smad signaling pathways [21]. Notably, MEF2A mRNA is highly expressed in skin tissue, and keratinocytes involved in wound healing account for up to 90% of human epidermal cells. However, the specific mechanisms by which MEF2A influences keratinocyte function and diabetic wound healing remained unclear. Our study not only confirmed the upregulation of MEF2A expression in db/db mouse diabetic wounds and HG‐induced HaCaT cells but also, more importantly, revealed for the first time that MEF2A overexpression effectively ameliorates HG‐induced keratinocyte dysfunction. This effect involves promoting proliferation and migration via regulation of the PI3K/AKT signaling pathway alongside inhibiting apoptosis.

The PI3K/AKT signaling pathway is well established as a regulator of numerous critical cellular functions, including proliferation, migration, adhesion, glucose metabolism, and angiogenesis [45]. PI3K, an intracellular phosphatidylinositol kinase, catalyzes the phosphorylation of PIP2 to generate the second messenger PIP3, which subsequently activates AKT. Activated AKT promotes cell survival and proliferation by phosphorylating a multitude of downstream targets, such as inhibiting pro‐apoptotic proteins like BAD and Caspase‐9, and regulating cell cycle‐related proteins like cyclins and CKIs [34, 42]. However, whether and how MEF2A influences keratinocyte function under HG conditions by promoting AKT phosphorylation has not been previously reported. Our study addresses this gap by elucidating the role of MEF2A‐PI3K/AKT axis in improving keratinocyte function under hyperglycemic conditions.

Nevertheless, this study has certain limitations. Although we have validated the expression pattern of MEF2A in diabetic mouse wound tissues in vivo, the mechanistic experiments were primarily conducted using the HaCaT cell line, which lacks the complexity of the in vivo microenvironment, including immune cell infiltration, impaired angiogenesis, and systemic metabolic disturbances. Diabetic wound healing involves the coordinated action of multiple cell types and systems, and a simple cell model cannot fully recapitulate the in vivo regulatory network of MEF2A under pathophysiological conditions such as hyperglycemia and chronic inflammation. Therefore, the effect of MEF2A requires further validation in diabetic mouse wound models. Second, as a transcription factor, the functional activity of MEF2A depends on its downstream target genes. It has been reported that HG induces the translocation of MEF2A from the nucleus to the cytoplasm [21]. This nucleocytoplasmic shuttling represents a rapid and efficient mechanism for regulating transcription factor activity, which has been widely observed in other proteins such as FOXO [46], NFAT [47], and p53 [48]. However, although this study confirmed that MEF2A regulated cellular functions via the PI3K/AKT pathway, the underlying molecular mechanisms remain to be fully elucidated, including whether such translocation also occurs in keratinocytes, the identity of its direct target genes, and the precise nature of its interaction with this pathway. Finally, this study focused on the HG environment and did not address other critical pathological factors in diabetic wounds, such as the accumulation of advanced glycation end products (AGEs) and oxidative stress. The role of MEF2A and its potential interactions with these factors require further investigation.

## 5 Conclusion

Diabetic wound healing is a refractory clinical complication of diabetes, and our study clarifies the critical role of MEF2A in regulating keratinocyte function during this process. We verified that MEF2A is upregulated in diabetic mouse wound tissues and HG‐treated HaCaT cells, and its overexpression improves HG‐induced keratinocyte dysfunction by activating the PI3K/AKT signaling pathway, which promotes keratinocyte proliferation and migration and inhibits apoptosis. This study is the first to uncover the regulatory mechanism of the MEF2A‐PI3K/AKT axis in diabetic wound healing, filling the research gap of MEF2A in this field and providing a novel potential therapeutic target for the clinical treatment of diabetic wounds.

## Author Contributions

Yuan Xue and Min Xuan contributed equally to this work as co‐first authors. Material preparation, data collection, and analysis were performed by Yuan Xue, Min Xuan, Linlin Feng, Jingwen Kuang, Bin Xu, and Limin Wei. The first draft of the manuscript was written by Yuan Xue and Min Xuan, and Pengcheng Xu contributed to the study conception and design, provided critical revision of the manuscript, and supervised the whole study. All authors commented on previous versions of the manuscript. All authors read and approved the final manuscript.

## Funding

This research was supported by the National Natural Science Foundation of China (82102338).

## Ethics Statement

All animal experiments were approved by the Ethics Committee of The First Affiliated Hospital of Henan University of Science and Technology (Approval Number: D‐2025‐B003) and conducted in accordance with the Guide for the Care and Use of Laboratory Animals.

## Conflicts of Interest

The authors declare no conflicts of interest.

## Supporting information


**Figure S1:** Validation of MEF2A overexpression effects on HaCaT cell function. (A) Cell viability evaluated by MTT assay (*n* = 3). (B, C) IF staining and quantitative results of Ki67 (red) and nuclei (DAPI, blue) (*n* = 3). Scale bar: 50 μm. (D) Cell apoptosis analyzed by flow cytometry (*n* = 3). (E) Representative images from wound healing (scale bar: 200 μm) assays showing HaCaT cell migration after 24 and 48 h treatment under the indicated conditions. Quantification of the wounded area closure rate (*n* = 3). Comparisons among three or more groups were conducted using one‐way analysis of variance, followed by Tukey's post hoc test. Data are presented as mean ± SD.

## Data Availability

The data supporting the findings of this study are available from the corresponding author upon reasonable request.
